# Supplementary material for: Dietary Pattern Associated with the Risk of Poor Glycemic Control in Chinese Diabetic Adults: Results from the China Nutrition and Health Surveillance 2015–2017 Survey
Source: Nutrients. 2022 Dec 23;15(1):56. doi: 10.3390/nu15010056 (PMC9824526; doi:10.3390/nu15010056)
Supplement: Supplementary file 1 [file nutrients-15-00056-s001.zip › nutrients-2077684-supplementary.pdf]

Supplementary Table S1. Recommendation of percentage of macronutrients intake from energy intake according to the *Guideline for the Prevention and Treatment of Type 2 Diabetes Mellitus in China (2020 edition).*

	**Low**	**Moderate**	**High**
**PECI**	<50%	50%~65%	>65%
**PEPI**	<15%	15%~20%	>20%
**PEFI**	<20%	20%~30%	>30%

PECI.: Percentage of energy from carbohydrate intake; PEPI.: Percentage of energy from protein intake; PEFI.: Percentage of energy from fat intake.
